# Change in HbA1c Levels between the Age of 8 Years and the Age of 12 Years in Dutch Children without Diabetes: The PIAMA Birth Cohort Study

**DOI:** 10.1371/journal.pone.0119615

**Published:** 2015-04-13

**Authors:** Hanneke Jansen, Alet H. Wijga, Salome Scholtens, Gerard H. Koppelman, Dirkje S. Postma, Bert Brunekreef, Johan C. de Jongste, Henriëtte A. Smit, Ronald P. Stolk

**Affiliations:** 1 University of Groningen, University Medical Center Groningen, Department of Epidemiology, Groningen, The Netherlands; 2 University of Groningen, University Medical Center Groningen, Department of Pediatric Pulmonology and Pediatric Allergology, Groningen, The Netherlands; 3 University of Groningen, University Medical Center Groningen, Department of Pulmonology, GRIAC Research Institute, Groningen, The Netherlands; 4 Centre for Nutrition, Prevention and Health Services, National Institute for Public Health and the Environment (RIVM), Bilthoven, The Netherlands; 5 The Institute for Risk Assessment Sciences, University of Utrecht, Utrecht, The Netherlands; 6 The Julius Centre for Health Sciences and Primary Care, University Medical Centre Utrecht, Utrecht, The Netherlands; 7 Department of Paediatrics, Erasmus MC-Sophia Children's Hospital, Rotterdam, The Netherlands; Virgen Macarena University Hospital, School of Medicine, University of Seville, SPAIN

## Abstract

**Objective:**

HbA1c is associated with cardiovascular risk in persons without diabetes and cardiovascular risk accumulates over the life course. Therefore, insight in factors determining HbA1c from childhood onwards is important. We investigated (lifestyle) determinants of HbA1c at age 12 years and the effects of growth on change in HbA1c and the tracking of HbA1c between the age of 8 and 12 years.

**Study Design and Methods:**

Anthropometric measurements were taken and HbA1c levels were assessed in 955 children without diabetes aged around 12 years participating in the PIAMA birth cohort study. In 363 of these children HbA1c was also measured at age 8 years. Data on parents and children were collected prospectively by questionnaires.

**Results:**

We found no significant association between known risk factors for diabetes and HbA1c at age 12 years. Mean(SD) change in HbA1c between ages 8 and 12 years was 0.6(0.7) mmol/mol per year (or 0.1(0.1) %/yr). Anthropometric measures at age 8 and their change between age 8 and 12 years were not associated with the change in HbA1c. 68.9% of the children remained in the same quintile or had an HbA1c one quintile higher or lower at age 8 years compared to age 12 years.

**Conclusion:**

The lack of association between known risk factors for diabetes and HbA1c suggest that HbA1c in children without diabetes is relatively unaffected by factors associated with glycaemia. HbA1c at age 8 years is by far the most important predictor of HbA1c at age 12. Therefore, the ranking of HbA1c levels appear to be fairly stable over time.

## Introduction

Several studies have shown an association between HbA1c and cardiovascular risk in people without diabetes [1,2] and it is known that cardiovascular risk accumulates over the life course. Since HbA1c is used as measure of the risk for cardiovascular complications, and may also be used for diagnosing diabetes [3], it is important to get better insight in the factors determining HbA1c from childhood onwards.

In a previous study we investigated determinants of HbA1c in 788 Dutch children without diabetes aged 8–9 years from the Prevention and Incidence of Asthma and Mite Allergy (PIAMA) birth cohort [4]. In the current study, we investigate anthropometric measures and life-style factors as determinants of HbA1c around the age of 12 years in the same children. In a subgroup of the study population, we investigate the change in and the tracking of HbA1c levels between the age of 8 and 12 years. Between the age of 8 and 12 years children go through a phase of important growth. The aim of the current study is to investigate the effects of this growth on the change in HbA1c levels and to investigate the stability of ranking of children by HbA1c over this 4 year period.

## Subjects and Methods

### Ethics Statement

This research was performed in accordance with the ethical principles for medical research involving human subjects outlined in the Declaration of Helsinki. The study protocol was approved by the Medical Ethics Committees of the participating institutes (Start project: Rotterdam MEC 132.636/1994/39 and 137.326/1994/130; Groningen MEC 94/08/92; Utrecht, MEC-TNO oordeel 95/50; Age 8 years: Utrecht, CCMO (Centrale Commissie Mensgebonden Onderzoek) P04.0071C, August 5th 2004; Utrecht, METC-protocol number 04–101 / K, July 27th 2004; Rotterdam, P04.0071C/MEC 2004–152, July 1st 2004; Groningen, 04.0071C/ M 4.019912, June 28th 2004; Age 12 years: Utrecht, METC (Medisch Ethische Toetsings-Commissie) protocol number 07–337 / K, May 20th 2008 07–337/K). All parents gave written informed consent.

### Design and study population

The study population consisted of 955 Dutch children born and recruited in 1996–1997 into the PIAMA birth cohort study in whom an HbA1c value was measured at the age of 12 years.

Details of the study design have been published previously [5]. Questionnaires were sent to the participating parents during pregnancy, at three months and yearly from 1 to 8 years of age and at 11 years of age. Details of the data collected during a hospital-based medical examination at 8 years of age have been described previously [4]. Around 12 years of age, all children who were still participating in the study were invited for a physical examination during a home-visit. In 964 children anthropometric measures were performed and an EDTA blood sample was taken. An HbA1c value could be assessed in 963 samples. Children with diabetes mellitus (n = 4) and children treated with growth hormone therapy (n = 2) were excluded from the analysis. Two children were excluded from the current analyses since they had unexplained high HbA1c levels of 6.4% (or 46 mmol/mol) and 6.8% (or 51 mmol/mol), possibly due to (still) unknown existence of diabetes mellitus or an analytic error. Finally, we included 955 children (Study population I). In 363 of these children HbA1c levels were also measured at the age of 8 years, in this subgroup we investigated the (determinants of) change in HbA1c (Study population II). Change in HbA1c was defined as the change in HbA1c in mmol/mol per year, to take into account the differences in the interval between both measurements between the children.

### Measures

For HbA1c analysis, erythrocytes were stored at -20°C for a mean period of 149 days (range 46–364) prior to assay. A 5 μl cell mass was lysated and HbA1c was measured by ion-exchange chromatography using the Adams A1c, HA-8160 HPLC (Menarini Diagnostics Benelux, Valkenswaard, The Netherlands). This analyser was standardised on Diabetes Control and Complications Trial (DCCT) standards. Between-batch imprecision (coefficient of variation) was 1.1% for a mean HbA1c of 5.9% and 0.8% for a mean HbA1c of 11.4%. Results were given as DCCT percentages as well as the new values of the International Federation of Clinical Chemistry and Laboratory Medicine (IFCC) in mmol/mol. New IFCC values were calculated with this equation: new IFCC value (mmol/mol) = (10.93 * old DCCT value)– 23.5 [6]. All HbA1c values were adjusted for storage time.

During the medical examination, children were weighed and measured in their underwear. Weight was measured to 0.1 kg and height to 0.1 cm by trained research staff using calibrated measuring equipment. Body mass index (BMI) was calculated as weight/height squared (kg/m^2^). ‘Overweight’ and ‘obesity’ were defined according to age and gender specific international standards [7]. We use the term ‘overweight’ for the group of children who are overweight but not obese. Waist-circumference, to the nearest 0.1 cm, was measured midway between the lowest rib and the top of the iliac crest at the end of gentle expiration. Hip-circumference, to the nearest 0.1 cm, was measured off the trochanter major. Waist-circumference as well as the hip-circumference was measured twice. The mean of the two measurements was used in the analysis. Standard deviation scores (SDS) of BMI, waist circumference, hip circumference and waist-to-hip circumference were calculated using Growth Analyser 3.5 (Growth Analyser B.V., Rotterdam, the Netherlands), based on Dutch reference values [8,9].

Data on diet were obtained from a food-frequency questionnaire filled out by the parents when the child was aged around 11 years. Consumption frequency was categorized for the intake of products with a high saturated fat content (i.e. butter, candy bars, fried snacks, fries, chips and chocolate) and high vitamin C content (i.e. fresh fruit, uncooked vegetables and cooked vegetables). In addition, the consumption frequency of fish and nuts was categorized in 3 categories. Data on physical activity were obtained from a questionnaire filled out by the children when they were around the age of 11 years. We calculated the time spent on walking or cycling to school and screen time (i.e. time spent on computing and watching television), in hours per week, and categorized both in 3 categories. In addition, we determined if they fulfilled the criteria of the Dutch physical activity guideline (at least one hour a day of active behaviour on every day of the week).

Besides assessing associations between a large number of different specific lifestyle items and HbA1c, we also considered the possibility that combinations of lifestyle factors might influence HbA1c levels. We therefore constructed three sum-scores: a saturated fat score (adding up the consumption category scores of products with a high saturated fat content), a healthy diet score (adding up the consumption category scores of fresh fruit, uncooked vegetables, cooked vegetables, fish and nuts) and a physical activity score (adding up the scores on the two physical activity indicators and subtracting the scores on screen time).

Data on maternal BMI and parental educational level (defined as the highest educational level of father and mother and categorized in three categories) were obtained by a questionnaire filled out by the parents when the children were 1 year old. Data on gestational diabetes were obtained by a questionnaire filled out by the parents of the children in whom an HbA1c levels was assessed at age 8 years.

### Statistical analyses

We used multiple linear regression, with only region and age at time of blood sampling as covariates (based on findings in the same population at age 8 years [4]), to investigate the relation of all separate potential determinants with the change in HbA1c and with HbA1c at 12 years. The associations between the three sum scores (‘high saturated fat’, ‘healthy diet’ and ‘physical activity’) and HbA1c levels at age 12 were assessed first in separate regression models for each of the scores individually and secondly by combining the three sum scores in one multivariable model.

To investigate the tracking of HbA1c over time, the classification of children by quintile of HbA1c at age 8 years was compared to the classification of children by quintile of HbA1c at age 12 years. A level of significance of p < 0.05 was applied for all analyses, which were performed with SPSS version 16.0 (SPSS Inc., Chicago, IL, USA).

## Results

The characteristics of the study populations are displayed in Table 1.

**Table 1:** Characteristics of the study population.

	*Study population I*			*Study population II*		
	Total n	Mean±SD	*n* (%)	Total n	Mean±SD	*n* (%)
***General***						
Gender: Girl	955		470 (49.2)	363		183 (50.4)
Age medical exam. 8yrs. (yrs.)	861	8.2 ± 0.4		363	8.1 ± 0.3	
Age medical exam. 12 yrs (yrs.)	955	12.5 ± 0.2		363	12.5 ± 0.2	
Δ Age (yrs.)	861	4.3 ± 0.5		363	4.4 ± 0.4	
***HbA1c***						
HbA1c at 8 yr. (mmol/mol)	363	29.8 ± 3.3		363	29.8 ± 3.3	
HbA1c at 12 yr. (mmol/mol)	955	32.2 ± 2.4		363	32.2 ± 2.4	
Δ HbA1c (mmol/mol)	363	2.4 ± 3.1		363	2.4 ± 3.1	
Change in HbA1c (mmol/mol/yr)	363	0.6 ± 0.7		363	0.6 ± 0.7	
HbA1c at 8 yr. (%)	363	4.9 ± 0.3		363	4.9 ± 0.3	
HbA1c at 12 yr. (%)	955	5.1 ± 0.2		363	5.1 ± 0.2	
Δ HbA1c (%)	363	0.2 ± 0.3		363	0.2 ± 0.3	
Change in HbA1c (%/yr)	363	0.1 ± 0.1		363	0.1 ± 0.1	
***Anthropometry at age 12 years***						
BMI (kg/m^2^)	955	18.8 ± 2.7		363	18.8 ± 2.6	
BMI SDS	955	0.19 ± 1.0		363	0.19 ± 1.0	
Δ BMI SDS	861	0.10 ± 0.6		363	0.07 ± 0.6	
BMI*	955			363		
Normal			829 (86.8)			316 (87.1)
Overweight			115 (12.0)			44 (12.1)
Obese			11 (1.2)			3 (0.8)
Waist circumference (cm)	955	66.2 ± 6.7		363	66.1 ± 6.4	
Waist circumference SDS	955	0.18 ± 1.0		363	0.18 ± 1.0	
Δ Waist circumference SDS	859	-0.19 ± 0.7		363	-0.22 ± 0.7	
Waist/hip ratio	955	0.82 ± 0.0		363	0.82 ± 0.0	
Waist/hip ratio SDS	955	0.07 ± 0.8		363	0.08 ± 0.8	
Δ Waist/hip ratio SDS	858	0.00 ± 0.8		362	0.00 ± 0.8	
***Parental factors***						
Maternal atopic constitution: Yes	955		303 (31.7)	363		229 (63.1)
Gestational diabetes: Yes	367	10 (2.7)		359	10 (2.8)	
Maternal BMI (kg/m^2^)	910	23.0 ± 3.4		345	23.1 ± 3.5	
Parental educational level	954			362		
Low		103 (10.8)			35 (9.7)	
Intermediate		344 (36.1)			130 (35.9)	
High		507 (53.1)			197 (54.4)	

Study population I: N = 955. HbA1c measured at the age of 12 years.

Study population II: N = 363. HbA1c measured at the age of 12 years and at the age of 8 years.

Δ = variable at age 12 years minus variable at age 8 years

* Defined according to age and gender specific international standards [7]

### Determinants of HbA1c at the age of 12 years

Table 2 shows associations between determinants and HbA1c levels at age 12 years. In a linear regression model, adjusting for region and exact age at blood sampling, HbA1c at age 8 years was significantly and positively associated with HbA1c at age 12 years, with an increase of 0.38 mmol/mol (95% CI 0.31–0.44) in HbA1c at 12 years per 1 mmol/mol increase in HbA1c at 8 years (or 0.38% (95% CI 0.31–0.44) per 1% increase)(p<0.001). The explained variance of this model was 28%. Children of mothers with gestational diabetes had significantly higher HbA1c levels at 12 years than their counterparts. HbA1c levels were not associated with parental education, not with overweight or obesity in the child and not with any of the other anthropometric measures. Out of 14 different lifestyle factors included in the analyses, only a few were significantly associated with HbA1c levels. HbA1c levels were significantly higher in children who consumed candy bars at least once a week than in non-consumers and significantly higher in children who adhered to the Dutch physical activity guideline than in children who did not adhere to the guideline. Intermediate consumption frequencies (as compared to low consumption frequencies) of cooked vegetables and of chocolate were associated with lower HbA1c levels, but for high consumption frequencies of vegetables and chocolate no associations were observed. The three lifestyle sum scores ‘high saturated fat’, ‘healthy diet’ and ‘physical activity’ were not associated with HbA1c levels. Also, when the three scores were included together in one multivariable model, no associations were observed and the model fit did not improve.

**Table 2:** Study population I. Determinants of HbA1c (mmol/mol) at the age of 12 years.

	HbA1c (mmol/mol)						
	n	Mean*	SD	Difference**	CI	p-value	R^2^ ***
Gender							0.04
Girls	470	32.2	2.5	-	-		
Boys	485	32.2	2.2	0.01	- 0.28–0.31	0.93	
BMI****							0.04
Normal	829	32.2	2.4	-	-		
Overweight	115	32.1	2.2	- 0.10	- 0.56–0.35	0.66	
Obese	11	32.4	3.2	0.24	- 1.15–1.63	0.73	
Gestational diabetes							0.04
No	357	32.1	2.3	-	-		
Yes	10	33.8	3.4	1.53	0.04–3.01	0.04†	
Parental educational level							0.04
Low	103	32.3	2.6	-	-		
Intermediate	344	32.2	2.4	- 0.10	-0.61–0.42	0.72	
High	507	32.2	2.3	- 0.04	-0.53–0.46	0.89	
Butter							0.04
No	669	32.2	2.4	-	-	-	
Yes	258	32.3	2.4	0.11	-0.23–0.44	0.53	
Candy bars							0.05
No	232	32.1	2.4	-	-		
< 1 day per week	522	32.2	2.3	0.10	-0.26–0.46	0.60	
≥ 1 day per week	173	32.7	2.4	0.57	0.11–1.03	0.02†	
Snacks							0.04
No	64	32.1	1.9	-	-		
< 1 day per week	646	32.2	2.4	0.09	-0.51–0.69	0.77	
≥ 1 day per week	217	32.5	2.4	0.35	-0.30–1.00	0.29	
Fries							0.04
No	437	32.3	2.2	-	-		
< 1 day per week	422	32.1	2.5	-0.16	-0.47–0.16	0.33	
≥ 1 day per week	68	32.3	2.5	-0.07	-0.67–0.53	0.82	
Chips							0.04
< 1 day per week	394	32.4	2.4	-	-		
1–2 day per week	463	32.1	2.4	-0.27	-0.59–0.04	0.09	
> 2 day per week	70	32.2	2.3	-0.22	-0.82–0.38	0.47	
Chocolate							0.04
0–1 day per week	197	32.5	2.5	-	-		
2–5 day per week	467	32.1	2.3	-0.44	-0.83 –-0.05	0.03†	
> 5 day per week	270	32.3	2.4	-0.21	-0.64–0.22	0.34	
Fresh fruit							0.04
< 3 day per week	130	32.4	2.2	-	-		
3–5 day per week	313	32.3	2.4	-0.00	-0.48–0.48	0.99	
> 5 day per week	484	32.2	2.4	-0.11	-0.56–0.35	0.65	
Uncooked vegetables							0.04
< 1 day per week	289	32.2	2.5	-	-		
1–2 day per week	344	32.3	2.3	0.06	-0.30–0.43	0.73	
> 2 day per week	294	32.3	2.3	0.11	-0.27–0.49	0.57	
Cooked vegetables							0.05
< 3 days per week	56	32.9	2.2	-	-		
3–5 days per week	508	32.1	2.3	-0.89	-1.53 –-0.25	0.01†	
> 5 days per week	362	32.4	2.4	-0.56	-1.22–0.09	0.09	
Fish							0.04
No	124	32.5	2.4	-	-		
< 1 day per week	454	32.2	2.4	-0.30	-0.77–0.16	0.20	
≥ 1 day per week	349	32.2	2.3	-0.29	-0.77–0.19	0.24	
Nuts							0.04
No	397	32.3	2.4	-	-		
< 1 day per week	423	32.1	2.3	-0.21	-0.53–0.11	0.21	
≥ 1 day per week	107	32.5	2.6	0.17	-0.33–0.67	0.50	
Active transport to school							
< 1 hours/week	356	32.3	2.3	-	-		0.04
1–1.5 hours/week	308	32.3	2.4	0.09	-0.26 – 0.45	0.61	
> 1.5 hours/week	268	32.1	2.5	- 0.17	-0.55–0.20	0.36	
Dutch physical activity guideline:							0.04
No	729	32.1	2.3	-	-		
Yes	205	32.6	2.5	0.42	0.05 – 0.78	0.03†	
Screen time							0.04
< 10 hours/week	398	32.3	2.3	-	-		
10–20 hours/week	377	32.2	2.4	- 0.18	-0.51 – 0.15	0.28	
> 20 hours/week	158	32.1	2.4	- 0.25	-0.68–0.18	0.25	
HbA1c at 8 years (mmol/mol)	363			0.38	0.31–0.44	<0.001§	0.28
Weight SDS	955			0.05	- 0.10–0.20	0.50	0.04
Height SDS	955			0.03	- 0.12–0.18	0.73	0.04
BMI SDS	955			0.05	- 0.10–0.19	0.52	0.04
Waist SDS	955			0.03	- 0.13–0.18	0.73	0.04
Hip SDS	955			0.04	- 0.12–0.21	0.60	0.04
Waist-to-Hip SDS	955			0.02	- 0.17–0.21	0.85	0.04
Maternal BMI (kg/m^2^)	910			0.02	- 0.03–0.06	0.47	0.04
Saturated fat score	923			0.02	-0.07–0.11	0.68	0.04
Healthy diet score	926			-0.10	-0.09–0.07	0.81	0.04
Physical activity score	929			0.06	-0.06–0.19	0.32	0.04

Study population I: N = 955. HbA1c measured at the age of 12 years.

* Crude mean

** Multiple linear regression model, only adjusted for age at medical examination 12 years and region

*** Explained variance

**** Defined according to age and gender specific international standards [7]

† P < 0.05

§ P < 0.001

### Change in HbA1c between the age of 8 and 12 years

The mean (SD) difference in HbA1c between the age of 8 and 12 years was 2.4 (3.1) mmol/mol (or 0.2 (0.3) %) (p<0.001). The mean (SD) increase was 0.6 (0.7) mmol/mol per year (or 0.1 (0.1) %/yr). In line with the observed association between HbA1c at ages 8 and 12 years, the ranking of HbA1c was fairly stable over time: 68.9% (n = 250) of the children remained in the same quintile (n = 114) or had an HbA1c level in one quintile higher (n = 70) or lower (n = 66) at age 8 years compared to age 12 years (Table 3).

**Table 3:** Number of children shifted between quintiles of HbA1c.

Δ Quintile	Frequency	%	Cumulative %
0	114	31.4	
1 or -1	136	37.5	68.9
2 or -2	66	18.2	87.1
3 or -3	40	11.0	98.1
4 or -4	7	1.9	100.0
Total	363	100.0	

Neither anthropometric measures at age 8 nor the change in anthropometric measure SD-scores per year between 8 and 12 years were associated with the change in HbA1c (Table 4). Also after adjustment for baseline anthropometric measure SDS (i.e. at age 8 years), the change in anthropometric measure SDS per year between 8 and 12 years was not associated with the change in HbA1c.

**Table 4:** Study population II. Determinants of the change in HbA1c (mmol/mol/yr).

	Change in HbA1c (mmol/mol/yr)					
	n	Mean*	SD	Difference**	CI	p-value
Gender						
Girls	183	0.6	0.7	-		
Boys	180	0.5	0.7	- 0.07	- 0.21–0.06	0.30
BMI 8 years***						
Normal	316	0.6	0.7	-		
Overweight	39	0.4	0.6	- 0.16	- 0.38–0.06	0.15
Obese	8	0.7	0.8	0.18	- 0.29–0.65	0.45
BMI SDS 8 years	363			- 0.05	- 0.13–0.02	0.17
Δ BMI SDS/yr	363			- 0.13	- 0.66–0.39	0.61
Waist circumference SDS 8 years	363			- 0.04	- 0.12–0.04	0.32
Δ Waist circumference SDS/yr	363			- 0.28	- 0.73–0.17	0.21
Hip circumference SDS 8 years	362			- 0.09	- 0.18–0.01	0.07
Δ Hip circumference SDS/yr	362			- 0.05	- 0.59–0.49	0.86
Waist/hip ratio SDS 8 years	362			0.04	- 0.06–0.14	0.40
Δ Waist/hip ratio SDS/yr	362			- 0.27	- 0.64–0.10	0.15
Maternal BMI (kg/m^2^)	345			0.00	- 0.02–0.02	0.85

Study population II: N = 363. HbA1c measured at the age of 12 years and at the age of 8 years.

* Crude mean.

** Multiple linear regression, only adjusted for age at medical examination 12 years and region.

*** Defined according to age and gender specific international standards [7]

## Discussion

We found no consistent significant association between life-style factors and HbA1c at the age of 12 years. HbA1c at age 8 years is by far the most important predictor of HbA1c at age 12. Therefore, the ranking of HbA1c levels appear to be fairly stable over time. We found a mean increase in HbA1c between age 8 and age 12 of 0.6 mmol/mol/yr (or 0.1%/yr). Anthropometric variables at age 8 years as well as change in anthropometry between age 8 and 12 were not associated with the change in HbA1c.

In our studies on determinants of HbA1c in children without diabetes around 8 years as well as 12 years of age, several (life-style) factors known to be associated with insulin resistance and risk for Type 2 diabetes, appear not to be associated with HbA1c. This is in line with the findings of Shultis et al. who concluded from their study to determinants of HbA1c in children that HbA1c is not a good marker of fasting or post-load glucose and insulin measures in healthy children, and that it is not a viable alternative to these measures for investigating the early life and childhood determinants of insulin resistance and Type 2 diabetes in children [10]. In contrast, other studies on determinants of HbA1c in childhood did find associations between know risk factors for Type 2 diabetes like overweight and parental history of diabetes and HbA1c [11–13]. Possibly, at this young age, the increased insulin resistance as a result of these risk factors presumably is not yet present or is fully compensated for by increased insulin production, resulting in normal glucose and HbA1c levels. Unfortunately, we did not assess insulin levels in our study.

In contrast to the studies described above, in the current study we were also able to investigate life-style factors, i.e. dietary intake and physical activity, as determinants of HbA1c. A higher physical activity level is known to decrease insulin resistance [14]. However, we found higher HbA1c levels in physically active children, both at age 8 years [4] and at age 12 years in the current study. Also, at both ages we found no consistent association between dietary factors and HbA1c, in contrast to studies in adults [15]. These results suggest that HbA1c in children without diabetes is relatively unaffected by level of glycaemia and factors associated with glycaemia. And that HbA1c is determined by life-style factors to a greater extent in adults compared to children or, alternatively, that the differences in life-style factors may be less among children than among adults. Further studies to investigate these differences are warranted. But the observed tracking found in this study is in line with this finding, suggesting that the factors causing between-individual variability of HbA1c in children without diabetes are fairly constant in an individual and therefore may not be environmental factors. At least not environmental factors we studied in the current study.

Puberty is associated with modest insulin resistance [16]. Given the mean (SD) age of our study population of 12.5 (0.2) small differences in pubertal development could be expected. Unfortunately, we had no good measure to determine the early pubertal development at the moment the blood sample was taken. Therefore, we were not able to investigate the influence of puberty on HbA1c levels.

Three studies previously reported that the ranking of HbA1c is relatively stable over time in adults [17–19]. Meigs et al. concluded, in their study on tracking of HbA1c over a period of 4–6 years, that HbA1c reliably categorizes the glucose control of subjects without diabetes over a period of 4–6 years, thereby confirming its value as an epidemiological measure. Our results are in line with these findings. But to our knowledge, we are the first who can confirm these findings in children. The stability of ranking of HbA1c over time suggests that the factors causing between-individual variability of HbA1c are fairly constant in an individual.

In the current study as well as in our study on determinants of HbA1c in children without diabetes around 8 years we found that HbA1c levels were significantly higher in the offspring of mothers with gestational diabetes compared with the offspring of mothers without gestational diabetes. Although the numbers are small, these data suggest that the offspring of mothers with gestational diabetes, so children with a genetic background of glucose intolerance, already have a relatively high HbA1c level at the age of 8 years and this relatively high HbA1c level is still present at the same extent at the age of 12 years. Probably, this genetic background of glucose intolerance is a constant factor determining HbA1c in children.

We investigated determinants of change in HbA1c in a subgroup of only 363 children. We found, though not statistically significant, unexpected inverse associations between the change in anthropometric measures and the change in HbA1c. The effect of especially the change in waist circumference SDS and waist-to-hip ratio SDS on the change in HbA1c seems to be quite large compared to the mean change in HbA1c. But this effect is, probably due to a lack of power, not significant. In the study population invited for a hospital-based medical examination at the age of 8 years, offspring of allergic mothers (mothers with asthma ever, allergy to house dust mite or pets or with hay fever) were oversampled [4]. Consequently, in our study population II also offspring of allergic mothers are overrepresented compared to study population I. We repeated all analyses in the offspring of allergic and non-allergic mothers separately and the results were largely the same in both groups.

In conclusion, we found no significant association between known risk factors for diabetes and HbA1c at the age of 12 years. HbA1c at age 8 years is by far the most important predictor of HbA1c at age 12 years and consequently, the ranking of HbA1c in children without diabetes seems to be fairly stable over time. These results suggest that HbA1c in children without diabetes is relatively unaffected by factors associated with glycaemia and that the factors causing between-individual variability of HbA1c are fairly constant in an individual.
